# Bioinformatics‐Driven Design of Fusion Recombinant Protein (EgFABP1‐EgTeg) for Enhanced Immunodiagnosis of Hydatid Cysts

**DOI:** 10.1002/iid3.70401

**Published:** 2026-05-12

**Authors:** Abolfazl Masoumi Koushk Mehdi, Hossein Motedayyen, Mohsen Arbabi, Amin Moradi Hasan‐Abad

**Affiliations:** ^1^ Department of Medical Parasitology, School of Medicine Kashan University of Medical Sciences Kashan Iran; ^2^ Autoimmune Diseases Research Center Kashan University of Medical Sciences Kashan Iran; ^3^ Health Information Management Research Center Kashan University of Medical Science Kashan Iran; ^4^ Infectious Diseases Research Center Kashan University of Medical Sciences Kashan Iran

**Keywords:** bioinformatics, EgFABP1, EgTeg, hydatid cyst, immunodiagnosis

## Abstract

**Background:**

Hydatid cyst disease is caused by the parasite *Echinococcus* and poses significant health concerns worldwide. Due to the lack of early symptoms and limited diagnostic tools, researchers aim to design a more specific and sensitive antigen. The study focuses on developing a recombinant multi‐epitope antigen using two parasite proteins (EgTeg and EgFABP1) and the IH4 nanobody.

**Methods:**

Protein sequences were analyzed and validated using bioinformatics tools, and B‐cell epitopes were identified. The resulting antigen, confirmed by UniProt, is 266 amino acids long.

**Results:**

The multi‐epitope antigen lacks a signal peptide and contains 46 phosphorylation sites associated with serine and tyrosine. Structural predictions showed both alpha helices and beta sheets in the secondary structure, with a spherical tertiary structure. Both linear and discontinuous epitopes were predicted, indicating regions with potential to stimulate immune responses. The antigen's physicochemical properties—molecular weight, isoelectric point stability index, and hydrophilicity—indicate that it is stable and suitable for diagnostic use.

**Conclusions:**

The study introduces the EgFABP1‐EgTeg‐IH4 recombinant protein as a promising candidate for diagnosing HC disease. By integrating multiple antigenic regions and the IH4 nanobody, this approach significantly improves diagnostic specificity and sensitivity, offering the potential for more accurate, earlier detection.

## 1 Introduction

Hydatid cyst (HC) disease, also known as cystic echinococcosis (CE), is a parasitic infection caused by the larval stage of the tapeworm *Echinococcus granulosus* [1]. The disease has become a major concern in many parts of the world, including Iran [2].

Cystic echinococcosis can be difficult to diagnose in the early stages because there are no pathognomonic signs [3]. The effectiveness of therapeutic strategies to cure CE would be greatly enhanced by early disease diagnosis [4]. Through circumvention, CE morbidity and mortality rates could be significantly reduced [5]. Diagnostic methods for CE detection include ultrasonography (US), computed tomography (CT), and magnetic resonance imaging (MRI), as well as serological tests, such as ELISA and haemagglutination [6, 7]. Furthermore, imaging alone makes it difficult to confirm the presence of larvae. However, the main serological method for diagnosing CE in humans is early detection of IgG antibodies [8]. In addition to this method's insufficiencies, it is also associated with poor prognostic value and low sensitivity and specificity [9].

The main problem with serological methods is cross‐reactivity with antigens of other parasites (e.g.,*Taenia solium*, *Taenia crassiceps*, *Toxocara canis*, *Schistosoma mansoni*) [10]. In combination with several specific antigens, fusion proteins may improve the efficacy of serological tests by providing multifunctional properties, which result in specific functions [11]. Recombinant antigens are widely used to detect a variety of diseases, including CE. With the help of these devices, cross‐reactions with other tapeworms would be diminished, and serological tests would become more specific and effective [12]. The hydatid cyst's antigen B is made up of various subsets with molecular weights of 8, 16, 24, and 32 kDa [13]. Novel antigens could be generated using Fusion protein technology to improve the performance of CE serodiagnostic methods [14]. Designing a novel diagnostic antigen would significantly enhance the fight against CE, given the diagnostic challenges of CE detection. Biological investigations have always relied on the in‐silico approach for designing novel diagnostic and therapeutic agents [15]. This novel antigen comprises three subunits: the EgTeg protein, the EgFABP1 protein, and an IH4 nanobody (VHH) [16, 17]. The EgTeg protein, with its multiple epitopes, has been identified as a suitable antigen for vaccine and diagnostic kit development. The presence of this protein in the larval stage, specifically within the protoscolex and cyst wall, has been confirmed using immunofluorescence [16, 18]. EgFABP1 protein has been confirmed in the protoscolex structure and throughout the parasite's life cycle. It functions as a protective antigen and plays a crucial role in the absorption of fatty acids, which are essential for parasite growth. However, this protein also stimulates the host's immune system, leading to the production of IgE antibodies [19]. Nanobodies, which are called VHH or IH4, are derived from heavy‐chain antibodies found in camels [20]. The specific binding of IH4 nanobody to glycophorin A on red blood cells makes it a suitable candidate for incorporation into a recombinant antigen [21]. This antigen can then be used to develop hemagglutination assays [17, 22].

In this study, we used an integrated in silico approach to develop a novel diagnostic antigen for detecting CE. Various bioinformatics tools have been used for structural and immunological analyses of EgTeg and EgFABP1‐IH4 from *E. granulosus*. The final diagnostic antigen capable of detecting CE infection was designed using the most immunogenic domains of each antigen.

## 2 Materials and Methods

### 2.1 The Amino Acid Sequences of Proteins

**Figure 1:**
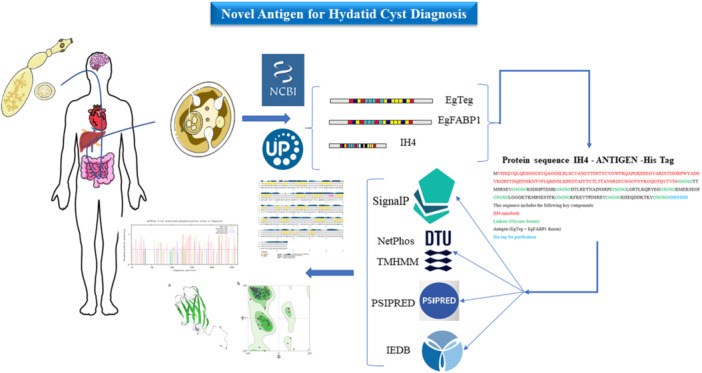
Abstract and overview of this research.

Protein sequences for EgTeg, EgFABP1 from *E*. *granulosus*, and IH4 (nanobody) antigens were obtained from the NCBI protein database (http://www.ncbi.nlm.nih.gov/protein). We confirmed the sequences of the proteins by searching UniProt (Figure 1) at (http://uniprot.org/). The protein sequences of the EgTeg, EgFABP1, and IH4 antigens were used for BLAST searches at (https://blast.ncbi.nlm.nih.gov/Blast.cgi) to identify conserved regions. By BLASTing against the EgTeg, EgFABP1, and IH4 sequences, we would identify the most similar sequences.

### 2.2 The Construction of Multi‐Epitope Antigen

To make the final antigen, we connected the linear epitopes of B cells to the proteins (EgTeg and EgFABP1) through the GPSL linker. Then we linked these epitopes to the IH4 nanobody via a linker in order to specifically bind to glycophorin A on red blood cells. The 3D structures of the constructed antigens were predicted using SwissModel software (Figure 1).

### 2.3 Signal Peptide of Multi‐Epitope Antigen

To determine whether the IH4 Antigen Histag contains a signal peptide, the protein's amino acid sequence was first selected and analyzed. Then, bioinformatics tools such as SignalP 5.0, developed by DTU Health Tech Bioinformatic Services, were used to predict the presence of signal peptides. The results were presented in tables and graphs, and the probability that a signal peptide was evaluated.

### 2.4 Transmembrane Helices of Multi‐Epitope Antigen

To predict membrane helices in proteins, the TMHMM bioinformatics tool (TMHMM 2.0, DTU Health Tech Bioinformatic Services), based on a hidden Markov model, was used. In this model, the possibility of passing through the cell membrane or inside the cell was investigated.

### 2.5 Phosphorylation Sites in Multi‐Epitope Antigen

First, the protein sequence was submitted to the NetPhos 3.1 server, and phosphorylation site predictions were generated. The analysis results included the total number of phosphorylation sites and their distribution across serine, threonine, and tyrosine residues. The data were presented as visual graphs showing the probability of phosphorylation at different points along the protein sequence.

### 2.6 The Secondary and Third Structure of Multi‐Epitope Antigen

Proteins IH4 ‐ ANTIGEN ‐HisTag secondary structures were analyzed using the PSIPRED online software (PSIPRED · bio. tools). Alpha helix, Extended strand, Beta turn, and Random coil structures were also analyzed. Using homology modeling, threading, and ab initio methods, the 3D structures of the three antigens were predicted. Protein 3D structure can be predicted most accurately through homology modeling. To evaluate the quality of model predictions for each antigen, we used the QMEAN software [23]. For complete structural refinement, the selected models were submitted to the 3DRefine software (http://sysbio.rnet.missouri.edu/3Drefine/).

### 2.7 B‐Cell Epitopes of Protein Fusion

Various B‐cell epitope prediction software were applied to analyze the 3D structures and sequences of IH4 ‐ ANTIGEN ‐HisTag to identify their linear and conformational epitopes. Linear epitopes, β‐turns, surface accessibility, flexibility, antigenicity, and hydrophilicity of IH4 ‐ ANTIGEN ‐HisTag were analyzed using IEDB online software (tools. iedb. org) and BepiPred (https://services.healthtech.dtu.dk/services/BepiPred-3.0/). Then, surface accessibility, antigenicity, flexibility, and hydrophilicity were analyzed using NetSurfP software.

### 2.8 The Prediction of Physicochemical Parameters of Proteins

The online software ProtParam (http://web.expasy.org/protparam/) was used to analyze the physicochemical properties of IH4 ‐ ANTIGEN ‐HisTag, including atomic composition, molecular weight, theoretical isoelectric point (PI), charge polarity, stability, hydrophobicity, etc.

## 3 Results

### 3.1 Proteins Sequences

#### 3.1.1 Tegumental Protein

The amino acid sequence of the tegumental protein (EgTeg) was extracted from the bioinformatics database and confirmed using the UniProt tool. The for Protein EgTeg version number is AAX20156.1. The amino acid sequence is as follows:

ATDPTTMSRSEVEVLKSDMPTEMKNFIIDQVDDTLREYNADSSRPIKLESLVTQLGRTLKQRYEGVWQVVILTGSYSAFSAYTPERLFHFKFGRFVVLVWQSSTY [16].

#### 3.1.2 Fatty Acid‐Binding Protein

The version of Protein EgFABP1 is Q02970.2, and the amino acid sequence is as follows:

MEAFLGTWKMEKSEGFDKIMERLGVDFVTRKMGNLVKPNLIVTDLGGGKYKMRSESTFKTTECSFKLGEKFKEVTPDSREVASLITVENGVMKHEQDDKTKVTYIERVVEGNELKATVKVDEVVCVRTYSKVA [16].

We confirmed the protein sequences by searching UniProt.

#### 3.1.3 The Amino Acid Sequence for IH4

The amino acid sequence for IH4 was obtained from US Patent No. US 9,879,090 B2, with corrections kindly provided by Olivier Bertrand. The sequence is as follows:

VHSQVQLQESGGGSVQAGGSLRLSCVASGYTDSTYCVGWFRQAPGKEREGVARINTISGRPWYADSVKGRFTISQDNSKNTVFLQMNSLKPEDTAIYYCTLTTANSRGFCSGGYNYKGQGTQVTVSS

#### 3.1.4 Protein Sequence IH4 ‐ Antigen ‐His Tag

The recombinant protein developed for the diagnosis of HC disease was constructed by fusing the EgTeg and EgFABP1 proteins with an IH4 nanobody. The amino acid sequences of these components were linked using glycine‐serine linkers. The detailed amino acid sequence, along with the separation of its components, is as follows: The amino acid sequence for the recombinant fusion protein, including the IH4 nanobody, linkers, and antigen components, is as follows:

MVHSQVQLQESGGGSVQAGGSLRLSCVASGYTDSTYCVGWFRQAPGKEREGVARINTISGRPWYADSVKGRFTISQDNSKNTVFLQMNSLKPEDTAIYYCTLTTANSRGFCSGGYNYKGQGTQVTVSSGSGSGTTMSRSEVGSGSGKSDMPTEMKGSGSGDTLREYNADSSRPIGSGSGLGRTLKQRYEGGSGSGKMEKSEGFGSGSGLGGGKYKMRSESTFKGSGSGKFKEVTPDSREVGSGSGKHEQDDKTKVGSGSGHHHHHH

This sequence includes the following key components:

IH4 nanobody

Linkers (Glycine‐Serine)

Antigen (EgTeg + EgFABP1 fusion)

His‐tag for purification

#### 3.1.5 Examining the Presence of Signal Peptide in Protein Fusion

**Figure 2:**
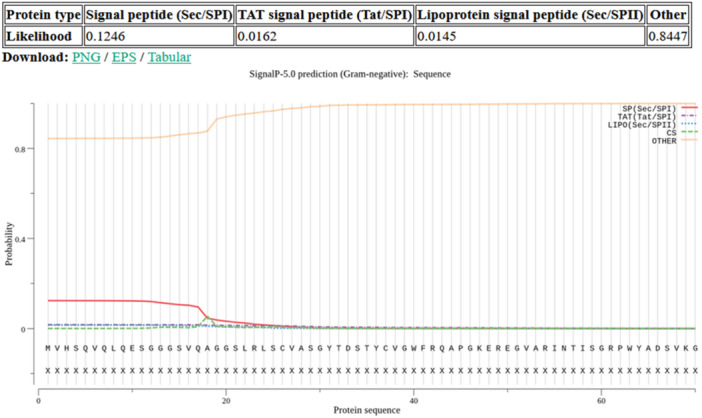
Signal peptide prediction of the recombinant protein. Computational analysis indicates a low probability of signal peptide presence, suggesting cytoplasmic localization.

Signal peptide analysis (Figure 2) revealed that the recombinant proteins expressed in *E. coli* lack detectable signal peptide sequences. As shown in the table and figure below, the probability of the presence of a signal peptide is very low. This finding indicates that these proteins are unlikely to be directed to the secretory pathway or the periplasmic space and instead remain in the cytoplasm. In *E. coli*, signal peptides are essential for targeting proteins to the secretory pathway, which can lead to their translocation across the inner membrane or secretion into the extracellular environment. The absence of a signal peptide suggests that the protein is retained in the cytoplasm, potentially limiting its access to specific cellular compartments or extracellular targets. Moreover, cytoplasmic localization may influence the protein's folding and stability, as certain post‐translational processes, such as disulfide bond formation, primarily occur in the periplasmic space in *E. coli*. This limitation can impact the functionality of the recombinant protein, especially if it requires such modifications for proper activity. To address these challenges, strategies such as adding a synthetic signal peptide to the construct or using specialized *E. coli* strains that support the desired modifications could be implemented to improve protein localization and function.

#### 3.1.6 Prediction of Transmembrane Helices of Proteins

**Figure 3:**
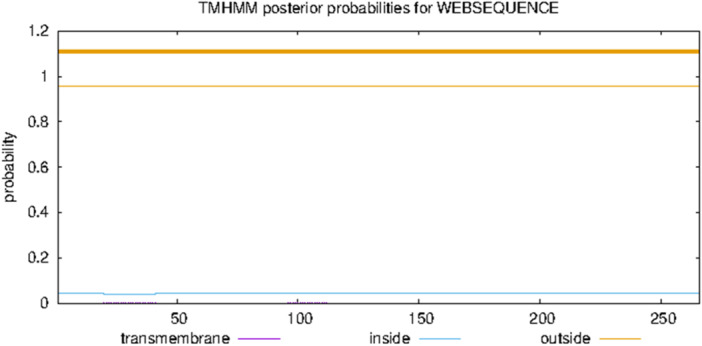
TMHMM posterior probability analysis for transmembrane regions. The purple line indicates transmembrane probability, and the orange line represents extracellular localization. Results suggest the protein is primarily non‐membrane‐associated.

TMHMM is a bioinformatics tool based on the Hidden Markov Model (HMM), widely used to predict transmembrane helices in integral membrane proteins. Analysis using this tool indicated that the IH4 Antigen Histag protein lacks a second transmembrane domain. As depicted in Figure 3, the purple line represents the posterior probability of transmembrane regions. In this analysis, the probability remains consistently close to zero, indicating the absence of transmembrane regions in the protein. The light blue line shows the likelihood of parts of the protein being localized inside the cell. This likelihood starts high but diminishes and eventually approaches zero, suggesting minimal intracellular presence.

On the other hand, the orange line indicates the likelihood that regions of the protein are localized outside the cell. This likelihood is close to 1 throughout the protein, strongly suggesting that it is predominantly extracellular. These findings indicate that the IH4 Antigen Histag protein is likely a secreted protein or resides on the cell surface without spanning the plasma membrane.

#### 3.1.7 Prediction of Phosphorylation Sites in Protein

**Figure 4:**
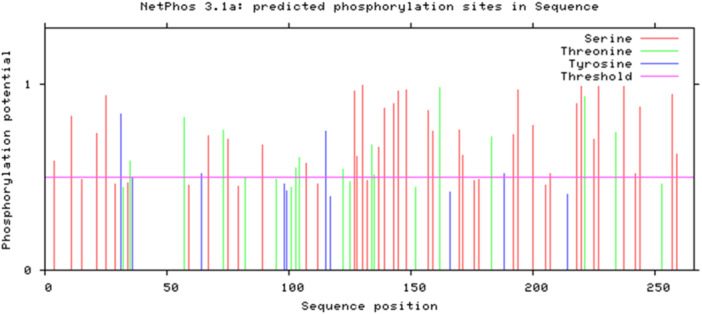
Predicted phosphorylation sites in the protein sequence. Serine, threonine, and tyrosine residues above the threshold line indicate potential PKC and PKA target sites.

The results from the NetPhos 3.1 online server analysis (Figure 4) revealed that the recombinant protein contains 46 phosphorylation sites distributed among serine, threonine, and tyrosine residues. These sites are primarily targeted by protein kinase C (PKC) and protein kinase A (PKA). Specifically, the analysis identified 32 serine, 10 tyrosine, and 4 threonine phosphorylation sites, indicating serine residues as the predominant phosphorylation targets. Figure 4 illustrates the predicted phosphorylation sites within the protein sequence. The X‐axis represents sequence positions ranging from 1 to 250, while the Y‐axis shows the phosphorylation potential. Colored lines indicate the likelihood of phosphorylation at specific amino acids: Red for serine, Blue for threonine, and Green for tyrosine. The purple horizontal line represents the threshold above which phosphorylation potential is considered significant. Data points above this line indicate regions with a high likelihood of phosphorylation, highlighting key residues and regions for further investigation. These findings provide valuable insights into potential regulatory hotspots within the protein, which may play critical roles in its function, interactions, and post‐translational modifications.

### 3.2 Prediction of the Secondary Structure of Antigens

**Figure 5:**
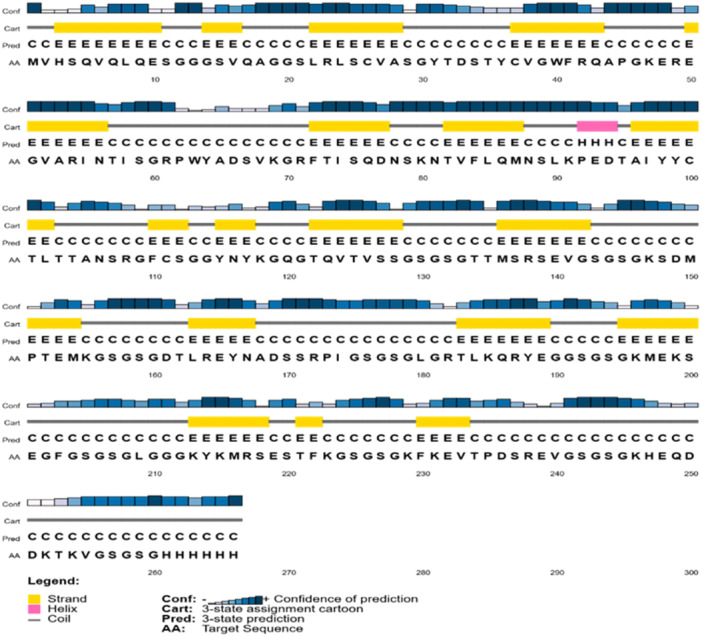
Predicted secondary structure and confidence of the protein sequence. Distribution of α‐helices, β‐strands, and coils is shown along with associated confidence levels.

Figure 5 illustrates the predicted secondary structure and the associated confidence levels of the protein sequence. The X‐axis represents sequence positions ranging from 1 to 300, with colored bars and symbols conveying structural information about each region. Blue bars indicate regions predicted to adopt an α‐helix structure, Yellow bars represent regions predicted to form β‐strands, White areas denote regions without a defined structure, and Gray bars indicate the confidence levels of the structural predictions. In each section, the symbols and colors reflect both the type of secondary structure and the confidence of the prediction for that specific region. Based on the analysis, the protein exhibits a diverse secondary structure, including significant portions of α‐helical and coil regions. The β‐strand structure is predominantly observed toward the end of the protein. Notably, the prediction confidence varies across different segments of the protein. Some regions are predicted with high confidence, indicated by robust gray bars, while other areas have lower confidence, represented by less prominent gray bars. This variability in confidence suggests that further experimental validation or more refined prediction tools may be necessary to develop a more accurate structural model.

### 3.3 3D Structure Prediction of Proteins and Ramachandran Diagram of This Protein

**Figure 6:**
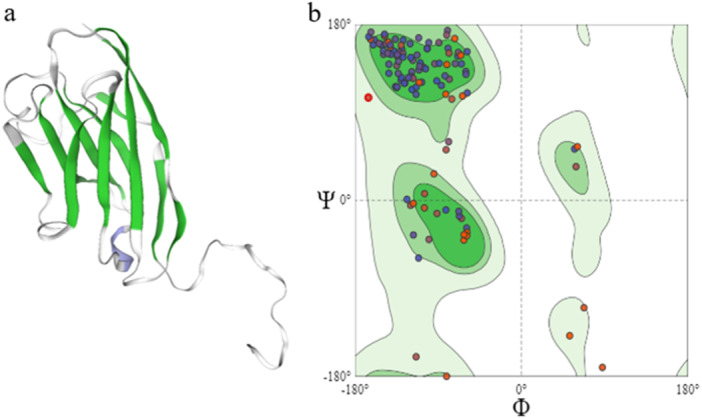
Tertiary structure of the protein. (a) The model highlights globular folding with predominant α‐helical domains. (b) Ramachandran plot showing the distribution of backbone dihedral angles (φ, ψ), confirming that the majority of residues lie in favored regions for α‐helices.

The predicted tertiary structure shows that the protein adopts a globular shape, with several helical domains. This overall structure is consistent with the secondary structure predictions shown in Figure 6, which indicate that α‐helices and coils are the major structural elements. The Ramachandran plot for the protein indicates that most of its amino acids are within the allowed regions for an α‐helical structure, supporting the observation of extensive helical regions in the tertiary model. Additionally, some amino acids are found in the allowed regions for β‐sheet structures, which aligns with the presence of multiple β‐sheet domains in the protein, as predicted in the secondary structure analysis. These findings suggest a well‐organized protein structure, predominantly α‐helical but also containing significant β‐sheet regions, which contribute to its overall globular conformation.

### 3.4 Model Assessment and Refinement

**Figure 7:**
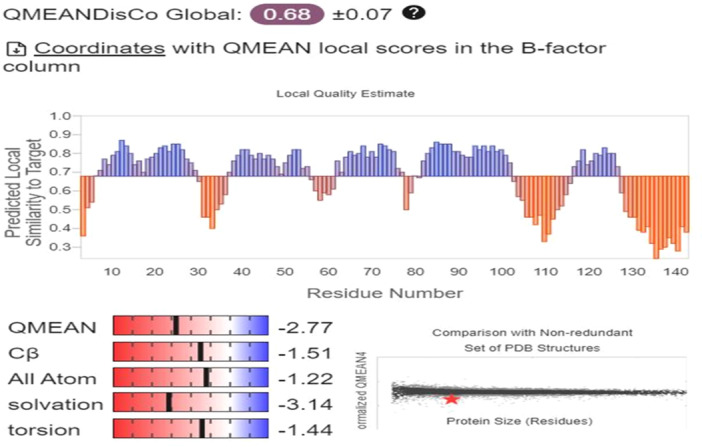
QMEAN‐based evaluation of the protein structural model. Regions of varying structural confidence are indicated based on comparison with known PDB structures.

Figure 7 presents the results of a qualitative evaluation of the protein structural model using the QMEAN method. QMEAN is a widely used tool for assessing the quality of protein structural models by comparing them to known protein structures in the Protein Data Bank (PDB). The overall quality of the structural model is considered average, with some regions exhibiting higher quality and others lower. This variability in quality suggests that while the model is generally reliable, certain areas may differ from known structures, indicating potential areas for improvement. These differences stem from limitations in the prediction method or inherent flexibility in the protein structure.

### 3.5 Linear and B‐Cell Epitopes

**Figure 8:**
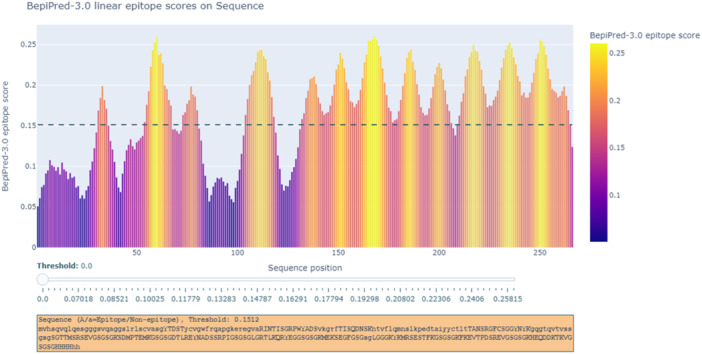
Prediction of linear B‐cell epitopes in the protein sequence using BepiPred 3.0. High‐probability regions are indicated.

This image shows linear epitope predictions for the protein sequence, generated by BepiPred 3.0. Epitopes are specific regions of an antigen recognized by the immune system, to which antibodies bind. Linear epitopes are contiguous sequences of amino acids that directly interact with antibodies. In Figure 8, the diagram highlights various regions along the protein sequence with a high probability of forming epitopes. These areas are represented as tall peaks on the graph, indicating the likelihood of antibody binding. By comparing the positions of these peaks with the protein sequence, specific amino acids likely to interact with the immune system can be identified. This information is crucial for understanding the protein's potential immunogenicity and can help design vaccines or therapeutic antibodies.

### 3.6 IEDB Analysis Resource

#### 3.6.1 Predicted Linear Epitope(s)

The table displays the results of B‐cell epitope prediction for the protein sequence, providing valuable insights into potential regions that may interact with the immune system. These epitopes, which range from 4 to 17 amino acids in length, are identified based on their likelihood of being recognized by B cells and triggering an immune response. Each predicted epitope is assigned a score reflecting its antigenicity, with higher scores indicating a greater likelihood of immunogenicity. The table lists the following information for each predicted epitope: Chain: Indicates the protein chain in which the epitope resides. Start and End: Show the position of the epitope within the protein sequence. Peptide: The actual amino acid sequence predicted to be an epitope. Number of residues: The length of the epitope in amino acids. Score: The predicted antigenicity score for the epitope. The predicted epitopes vary in their antigenicity, with the highest‐scoring epitopes likely to be the most immunogenic. For example, the epitope “SSGSGSGTTMSRSEVGS” (residues 127–143) has the highest score of 0.839, suggesting it is the most likely to trigger an immune response.

**Figure 9:**
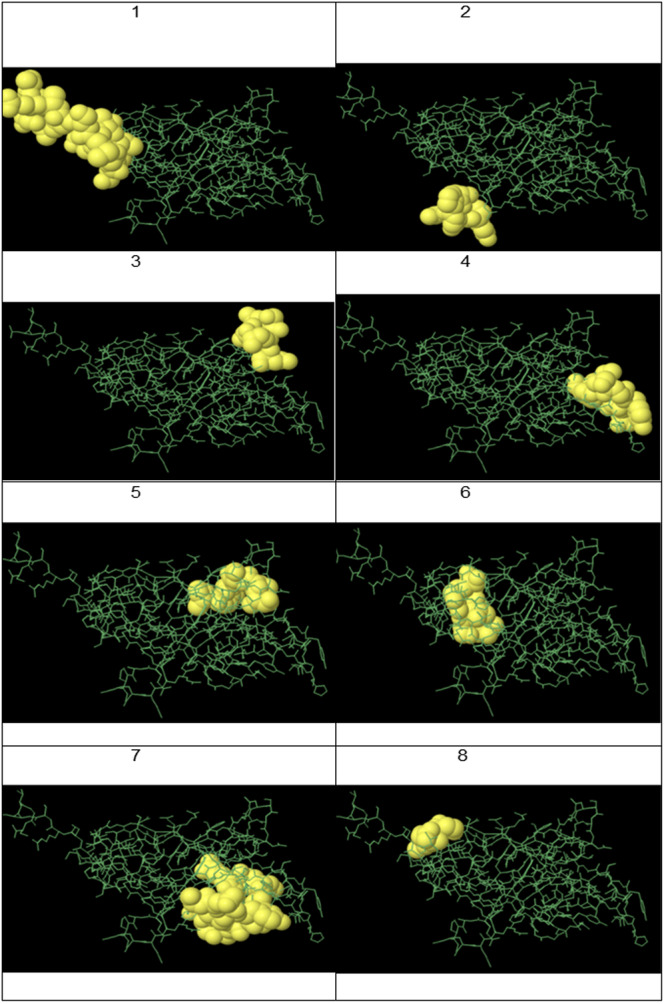
3D representation of predicted linear B‐cell epitopes. Yellow regions indicate predicted epitopes; green lines represent the overall protein structure.

The results from Table 1 are visually represented in Figure 9, which shows a three‐dimensional model of the protein with the 8 predicted B‐cell epitopes highlighted. In this figure, the green lines depict the overall 3D structure of the protein, allowing us to understand better the spatial arrangement of the epitopes relative to other parts of the protein. The yellow areas in the figure represent the predicted epitopes, regions most likely to trigger an immune response. These areas are visually emphasized to highlight their importance. The length and shape of these epitopes vary, reflecting the diversity of their structures and characteristics. By comparing the 3D structure in Figure 8 with the data in Table 1, we can easily identify which regions of the protein are most likely to elicit an immune response. For example, epitopes with higher scores, such as “SSGSGSGTTMSRSEVGS” (score 0.839), are highlighted in the 3D model, suggesting their significant potential for immunogenicity. The prediction scores in the table provide a quantitative evaluation of these regions, helping prioritize the most likely and relevant epitopes for further investigation.

**Table 1:** Results of the prediction of B cell epitopes for protein sequence.

No.	Chain	Start	End	Peptide	Number of residues	Score
1	B	127	143	SSGSGSGTTMSRSEVGS	17	0.839
2	B	44	48	APGKE	5	0.807
3	B	77	81	DNSKN	5	0.807
4	B	29	36	SGYTDSTY	8	0.699
5	B	57	62	TISGRP	6	0.675
6	B	65	71	ADSVKGR	7	0.607
7	B	105	121	ANSRGFCSGGYNYKGQG	17	0.541
8	B	17	20	QAGG	4	0.505

*Note:* This table lists the predicted epitopes, their lengths, and corresponding antigenicity scores, which vary across different regions of the protein sequence. The higher the score, the greater the likelihood of the epitope being immunogenic.

#### 3.6.2 Predicted Discontinuous Epitope(s)

Table 2 presents the discontinuous epitopes identified in the recombinant protein. Discontinuous epitopes are regions where the amino acids involved are not adjacent in the primary sequence but are brought together in the protein's three‐dimensional structure, where the immune system can recognize them as a cohesive unit. The table identifies several potential discontinuous epitopes, with varying lengths and scores. The amino acids forming each epitope are spread across different parts of the sequence, highlighting their non‐contiguous nature. The scores assigned to each epitope indicate the predicted strength of its ability to elicit an immune response, with higher scores suggesting a greater potential for immune recognition. For example, the epitope comprising residues R138, S139, E140, V141, G142, and S143 (score 0.969) is highly likely to elicit an immune response. In contrast, other epitopes, such as A65, D66, S67, V68, and K69 (score 0.607), are predicted to have a weaker response.

**Table 2:** Discontinuous epitopes in recombinant protein.

No.	Residues	Number of residues	Score
1	B:R138, B:S139, B:E140, B:V141, B:G142, B:S143	6	0.969
2	B:D77, B:N78, B:S79, B:K80, B:N81	5	0.807
3	B:A44, B:P45, B:G46, B:K47	4	0.789
4	B:S127, B:S128, B:G129, B:S130, B:G131, B:S132, B:G133, B:T134, B:T135, B:M136, B:S137	11	0.768
5	B:H3, B:S4, B:Q5, B:V6, B:Q7, B:S29, B:G30, B:Y31, B:T32, B:D33, B:S34, B:T35, B:Y36, B:T57, B:I58, B:S59, B:G60, B:R61, B:P62, B:T104, B:A105, B:N106, B:S107, B:R108, B:G109, B:F110, B:C111, B:S112, B:G113, B:G114, B:Y115, B:N116, B:Y117	33	0.665
6	B:A65, B:D66, B:S67, B:V68, B:K69	5	0.607

*Note:* This table lists the discontinuous epitopes, their corresponding amino acid residues, the number of residues, and predicted scores. The epitope scores suggest varying strengths of immune response, with some epitopes likely to be more immunogenic than others.

**Figure 10:**
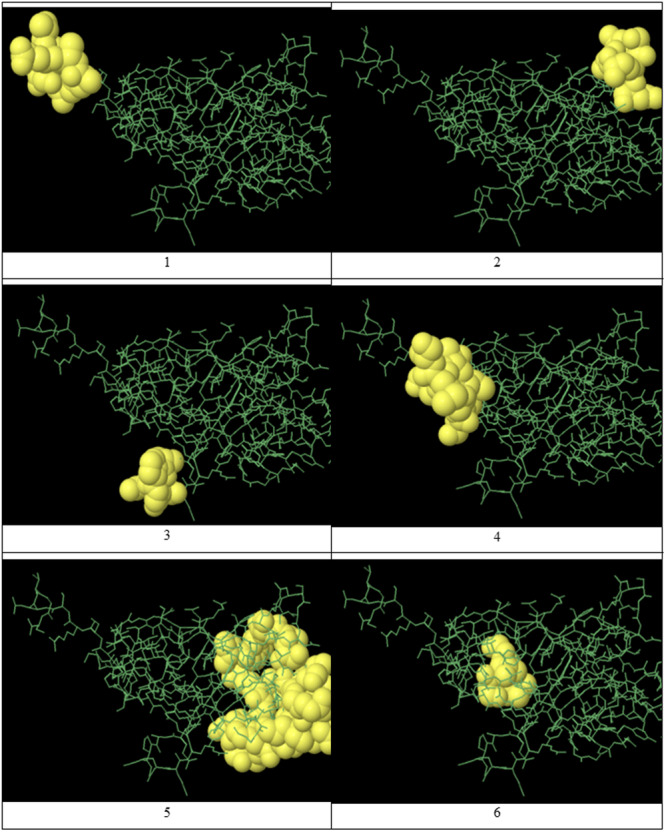
3D representation of predicted discontinuous B‐cell epitopes. Spatial arrangement of epitopes corresponds to Table 2.

Figure 10 visually represents the discontinuous epitopes identified in Table 2. This image helps visualize the spatial arrangement of these epitopes within the three‐dimensional structure of the recombinant protein, highlighting the specific locations where non‐contiguous amino acids come together to form functional epitopes. Comparing the image with the information in Table 2 shows that the identified discontinuous epitopes are distributed across different regions of the protein sequence. Yet, they are positioned close together in the 3D structure, making them potential targets for immune recognition. The graphical representation in Figure 9 confirms that these epitopes are strategically placed within the protein, suggesting they could play an important role in eliciting a strong immune response.

### 3.7 Prediction of Protein Surface Amino Acids

**Figure 11:**
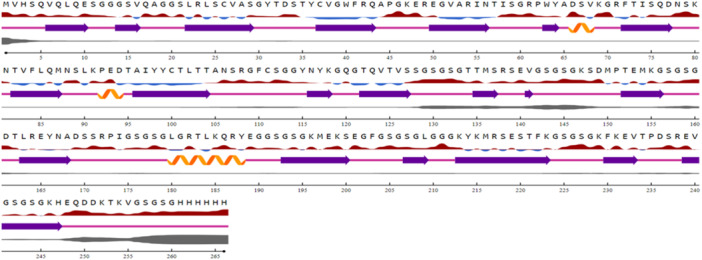
Predicted surface accessibility of amino acids in the protein. Color variations represent different levels of surface exposure.

Figure 11 presents a graphical representation of the protein's amino acid sequence, showing the probability of each amino acid being exposed on the protein surface. This prediction is based on algorithms that assess the likelihood of amino acids being accessible to interact with other molecules, such as proteins or ligands. In the diagram, colors indicate varying levels of surface accessibility. Lighter colors indicate higher accessibility, suggesting that those amino acids are more likely to be on the protein's surface. Conversely, darker colors indicate lower accessibility, meaning those amino acids are more likely to be buried inside the protein structure. Amino acids that are exposed on the surface are typically hydrophilic, while those buried inside the protein tend to be hydrophobic. Surface‐exposed amino acids are crucial for protein‐protein interactions and protein‐ligand binding, making this information valuable for understanding a protein's functional sites.

### 3.8 Physicochemical Properties of Final Antigen

The physicochemical properties of the recombinant IH4‐ANTIGEN‐HisTag protein are summarized in Table 3.

**Table 3:** Physicochemical properties of IH4‐ANTIGEN‐HisTag.

Property	Value	Interpretation
Number of amino acids	266	—
Molecular weight (Da)	27,838.38	—
Theoretical pI	9.27	Basic protein
Amino acid composition	Ala (3.0%), Arg (5.3%), Asn (2.6%), Asp (3.8%), Cys (1.5%), Gln (4.1%), Glu (5.3%), Gly (18.4%), His (3.0%), Ile (1.9%), Leu (3.8%), Lys (6.8%), Met (2.6%), Phe (2.6%), Pro (2.3%), Ser (16.2%), Thr (7.1%), Trp (0.8%), Tyr (3.8%), Val (5.3%)	Shows enrichment in Gly and Ser
Negatively charged residues (Asp + Glu)	24	—
Positively charged residues (Arg + Lys)	32	—
Atomic composition	C:1172, H:1844, N:362, O:405, S:11	Formula C1172H1844N362O405S11
Total number of atoms	3794	—
Extinction coefficient (M⁻¹ cm⁻¹, 280 nm)	26,150 (cystine), 25,900 (reduced)	Used for concentration estimation
Abs 0.1% (= 1 g/L)	0.939 (cystine), 0.930 (reduced)	—
Estimated half‐life	30 h (mammalian cells, in vitro); > 20 h (yeast, in vivo); > 10 h (*E. coli*, in vivo)	Indicates reasonable stability
Instability index (II)	37.11	Stable (< 40)
Aliphatic index	40.26	Reflects protein stability
GRAVY	−0.83	Hydrophilic, soluble

The IH4 ‐ ANTIGEN ‐HisTag protein consists of 266 amino acids and contains 3794 atoms. Its molecular formula is C_1172_H_1844_N_362_O_405_S_11_, and its molecular weight is 27.83 kDa. Theoretical calculations of the protein's isoelectric point (pI) indicate a value of 9.27, suggesting that the protein is basic. The protein contains 24 acidic residues (Asp + Glu), which are negatively charged, and 32 basic residues (Arg + Lys), which are positively charged. This balance of charged residues contributes to its overall charge at different pH levels. The instability index (II) of the protein was calculated to be 37.11. Since the instability index is below 40, the protein is predicted to be stable. A value above 40 would indicate a tendency toward instability. The aliphatic index, which indicates the volume occupied by aliphatic side chains (important for protein stability in various environments), is 40.26. The Grand Average of Hydropathicity (GRAVY) value is −0.83, indicating that the protein is hydrophilic. Proteins with a negative GRAVY value are generally water‐soluble and interact favorably with water, suggesting that the IH4 ‐ ANTIGEN ‐HisTag is soluble and likely interacts well in aqueous environments.

## 4 Discussion

Hydatid Cyst (HC) disease, also known as Cystic Echinococcosis (CE), is a parasitic infection primarily caused by *E. granulosus* and is recognized as a significant public health issue in many regions worldwide, including Iran [24]. As it is asymptomatic in the early stages of infection, CE can have a significant economic impact, particularly given veterinary treatment costs and its high prevalence among livestock [25]. Current diagnostic methods include imaging techniques such as sonography, computed tomography (CT), and magnetic resonance imaging (MRI), as well as serological tests like enzyme‐linked immunosorbent assay (ELISA) and agglutination tests [26, 27]. These methods frequently face challenges related to sensitivity, specificity, and cross‐reactivity with antigens from other parasites [28]. Therefore, the development of recombinant antigens capable of eliciting specific immune responses could improve the accuracy and efficiency of serological diagnosis of hydatid cysts [29]. It is imperative to diagnose CE early to avoid negative outcomes. Our in silico approach has therefore allowed us to design a novel diagnostic antigen that can circumvent preexisting diagnostic insufficiencies.

The antigen in this study comprised the key proteins EgTeg and EgFABP1, in combination with the nanobody IH4 [21, 30]. An EgTeg protein is a potential vaccine candidate. In the early stage of infection, this protein can significantly inhibit neutrophil chemotaxis. It is probably related to parasite survival [31]. It has already been confirmed that EgFABP1 can be expressed at the protoscolex and throughout the life of *E. granulosus*. This antigen is complete and inhibits the absorption of essential fatty acids required for parasite growth. As well as stimulating the production of specific antibodies, it can also stimulate the production of IgE antibodies [19]. These recombinant proteins have been optimized using bioinformatics methods to incorporate more immunogenic epitopes, thereby enhancing the sensitivity and specificity of diagnostic tests. To minimize the antigen size and enhance epitope accessibility, only the essential epitope‐bearing regions of EgTeg and EgFABP1 were included in the final antigen construct [32]. Previous studies have highlighted antigens such as AgB and EG95 as promising vaccine and diagnostic candidates [33, 34, 35, 36]. In this context, the present bioinformatics‐driven design of EgFABP1–EgTeg–IH4 fusion protein provides an additional step toward identifying effective recombinant antigens for CE diagnosis. Similar bioinformatics‐based studies have demonstrated the potential of other E. granulosus proteins, such as EgMyophilin, which has been predicted to contain multiple stable, non‐allergenic, and highly antigenic B‐cell and HLA‐binding epitopes, suggesting its promise as a vaccine candidate against CE [37]. The epitopes of this antigen, including four B‐cell epitopes and five HLA‐binding epitopes, had the highest antigenic index in the protein sequence [37]. Additionally, recent reviews have emphasized the diverse immunological aspects of CE and highlighted the importance of host immune responses in shaping diagnostic and vaccine strategies [33].

Prediction of B‐cell epitopes is one of the most significant findings of this study, as epitopes, which are parts of antigens recognized by the immune system, play a crucial role in eliciting an immune response. Results from epitope analysis revealed that this recombinant protein possesses both linear and discontinuous epitopes capable of inducing strong and specific immune responses [26].

The use of advanced technologies, such as 3D modeling and protein structure prediction, has also played a significant role in improving the design of recombinant antigens. For instance, 3D protein structure modeling enables a more detailed examination of molecular interactions, thereby enhancing the diagnostic efficiency of antigens. One of the most significant advantages of using recombinant antigens is the substantial reduction in cross‐reactivity, which is commonly observed in the serological diagnosis of hydatid cysts (CE). This feature is particularly important in regions where people may be infected with multiple parasite types [38].

It is worth mentioning that this recombinant protein has recently been experimentally evaluated in a separate study, demonstrating its diagnostic applicability in a novel direct hemagglutination test for CE [39]. These results provide preliminary validation of the bioinformatics‐driven design presented here.

## 5 Conclusion

In conclusion, the design and construction of the EgFABP1‐EgTeg‐IH4 recombinant fusion protein represent a promising step towards improving the serological diagnosis of HC disease. The integration of multiple antigenic domains and the IH4 nanobody has the potential to enhance the specificity and sensitivity of CE detection, addressing the limitations of existing serological methods. The findings of this study provide a solid basis for future experimental validation and further optimization of this diagnostic tool, which could contribute to the effective management and control of CE. One potential limitation of the current study is the lack of experimental validation of the recombinant fusion protein's diagnostic performance. While the bioinformatics analysis provides a strong foundation for the design and characterization of the antigen, additional in vitro and in vivo studies are needed to assess its sensitivity, specificity, and overall diagnostic accuracy.

## Author Contributions

**Abolfazl Masoumi Koushk Mehdi:** methodology, investigation, writing – review and editing. **Hossein Motedayyen:** writing – review and editing. **Mohsen Arbabi:** writing – review and editing, methodology, investigation, funding acquisition, supervision. **Amin Moradi Hasan‐Abad:** writing – review and editing, methodology, investigation, funding acquisition, supervision.

## Conflicts of Interest

The authors declare that there is no conflict of interest regarding the publication of this paper.

## Data Availability

The data that support the findings of this study are available in the supporting material of this article. All data generated or analyzed during this study are included in this published article and its supporting information file.
